# Supplementary figures and images for: Cellular and molecular characterization of peripheral glia in the lung and other organs
Source: PLoS One. 2024 Dec 2;19(12):e0310303. doi: 10.1371/journal.pone.0310303 (PMC11611111; doi:10.1371/journal.pone.0310303)

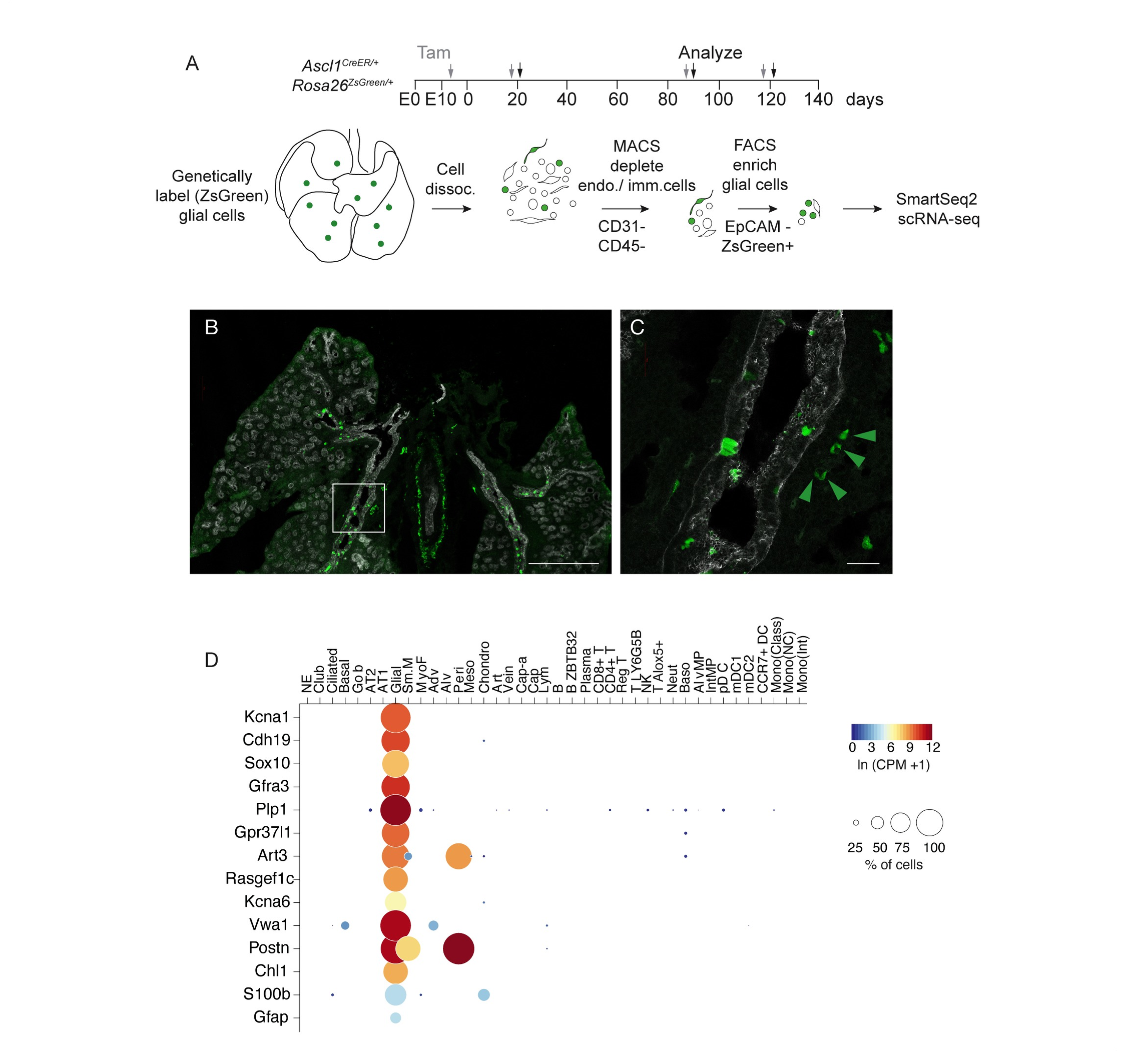

Supplement: S1 Fig — (A) Strategy for labeling and isolating lung glial cells using Ascl1CreER; Rosa26LSL-Zsgreen mice using a similar approach we previously reported [17]. Glial cells were enriched by sorting for lineage-labeled EpCAM—/ ZsGreen+ cells by fluorescence-activated cell sorting (FACS). (B) Lower-magnification confocal image of a section (35 μm thick) through an entire mouse lung lobe at E15.5 with glial and neuroendocrine cell progenitors lineage-labeled using (Ascl1 > ZsGreen1). Tamoxifen induction between embryonic (E) day 12.5-E13.5 and analysis at E18. E-cadherin (ECAD, white). Ascl1 –lineage labeled cells (Ascl1 > Zsgreen1, green). Scale bar, 200 μm. (C) Close-up view of boxed region in panel B. Four glial cell progenitors (green arrowheads) are located adjacent to airways. Neuroendocrine cells within the airway epithelium (ECAD+ regions) are also labeled because they selectively express Ascl1 within the epithelium. Scale bar, 20 μm. (D) scRNA-seq dot plot showing mean level of expression (dot intensity) and percent of cells in population with detected expression (dot size) of top glial markers across entire mouse atlas [15]. Cell types: NE, neuroendocrine (PNEC); AT1, alveolar epithelial cell, type 1; AT2, alveolar epithelial cell, type 2; Sm.M, smooth muscle; MyoF, myofibroblast; AdvF, adventitial fibroblast; AlvF, alveolar fibroblast; Peri, pericyte; Meso, mesothelial; Chondro, chondrocyte; Cap-a, capillary aerocyte; Cap, general capillary (Cap-g); Lym, lymphatic cell; B ZBTB32, B cells (ZBTB32+); Reg T, T cells (regulatory); T LY6G5B, T cells (LY6G5B+); NK, natural killer; T Alox5+, T cells (Alox5+); Neut, neutrophil; Baso, basophil; AlvMP, alveolar macrophage; IntMP, interstitial macrophage; pDC, plasmacytoid dendritic; mDC1, myeloid dendritic, type 1; mDC2, myeloid dendritic, type 2; CCR7+ DC, dendritic cell (Ccr7+); Mono(Class), monocyte (classical); Mono(NC), monocyte (non-classical); Mono (Int), monocyte (intermediate). (TIF) [file pone.0310303.s001.tif]

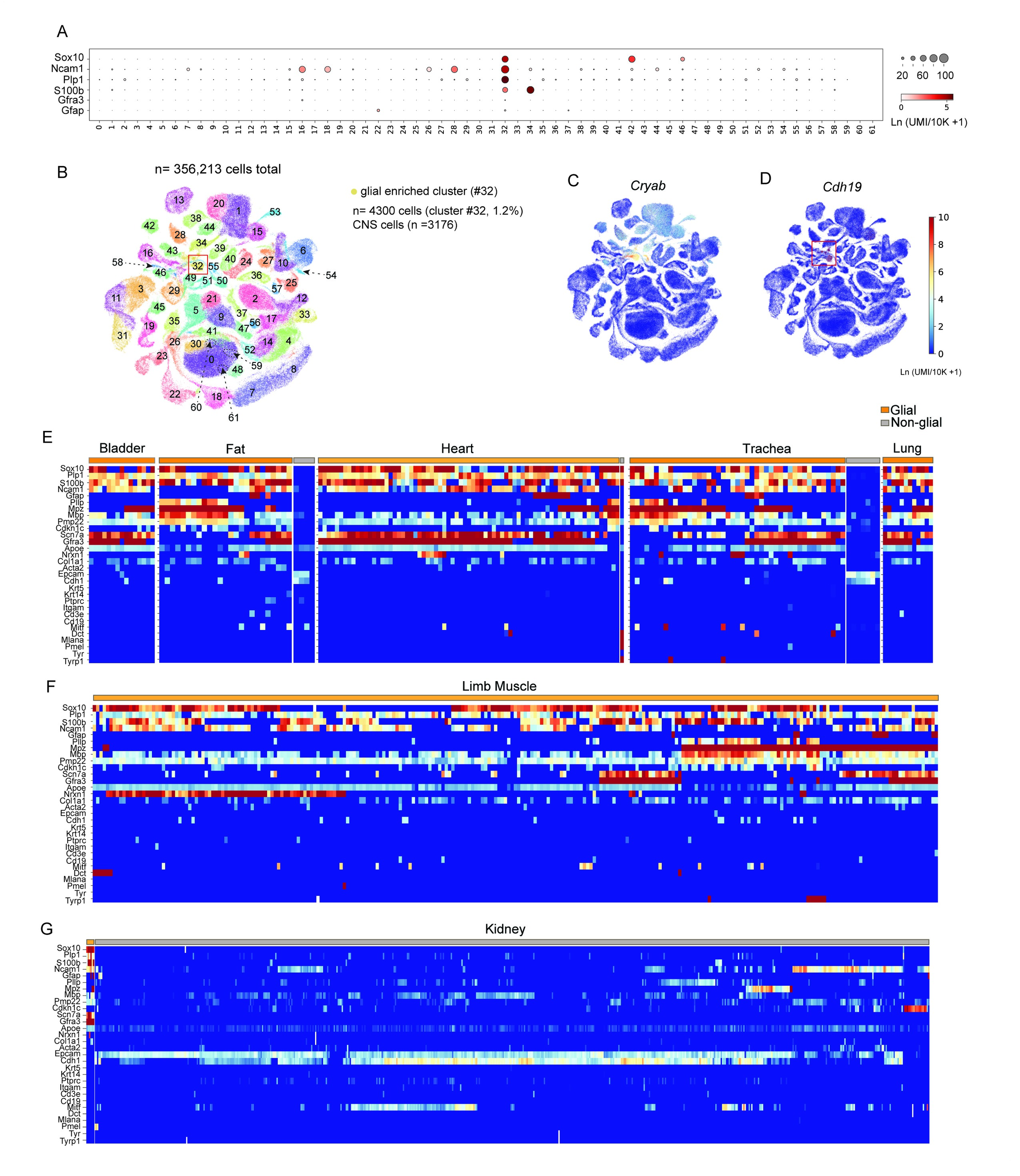

Supplement: S2 Fig — (A) Dotplot showing expression of classic glial markers (Sox10, Ncam1, Plp1, S100b, Gfra3, and Gfap) across all cell clusters in Tabula Muris Senis. Scale, Log-transformed unique molecular identifiers per 10,000 (ln (UMI)/10K +1). Size of circle indicates fraction of cells in each cluster expressing gene. Scale for all feature plots and heatmaps in this figure represent log-transformed unique molecular identifiers/10,000 +1 [ln (UMI/10K+1)]. (B) UMAP (uniform manifold approximation and projection) plot showing overview of the entire Tabula Muris Senis dataset. Individual clusters are numbered and colored as previously reported for the Tabula Muris Senis dataset (Tabula Muris Consortium, 2020 Nature). Boxed region (red rectangle) indicates cells in a single cluster (cluster #32, n = 4300 cells (1.2%) highly enriched for multiple classic glial genes (C–D) Expression of glial cell selective genes (Cryab and Cdh19) were identified by marker analysis (see methods section). Central nervous system (CNS) glial cells (n = 3176 cells) were removed from subsequent analysis. Scale, expression level. (E-G) Heatmaps showing expression of pan glial genes (Sox10, Plp1, S100b, Ncam1), the classic glial marker gene (Gfap), myelinating genes (Pllp, Mpz, Mbp, Pmp22, Cdkh1c) and non-myelinating genes (Scn7a, Gfra3, Apoe, Nrxn1) across each of the 7 organs in Tabula Muris in which we identified glial cells (indicated by light orange color bars above heatmap). Non-glial cells (grey color bar) were removed from the data set for a final group of 435 glial cells. Note that most of the kidney cells (G) expressed epithelial genes, Epcam and Cdh1, but not pan-glial genes. Thus, they were removed from further analysis. (TIF) [file pone.0310303.s002.tif]

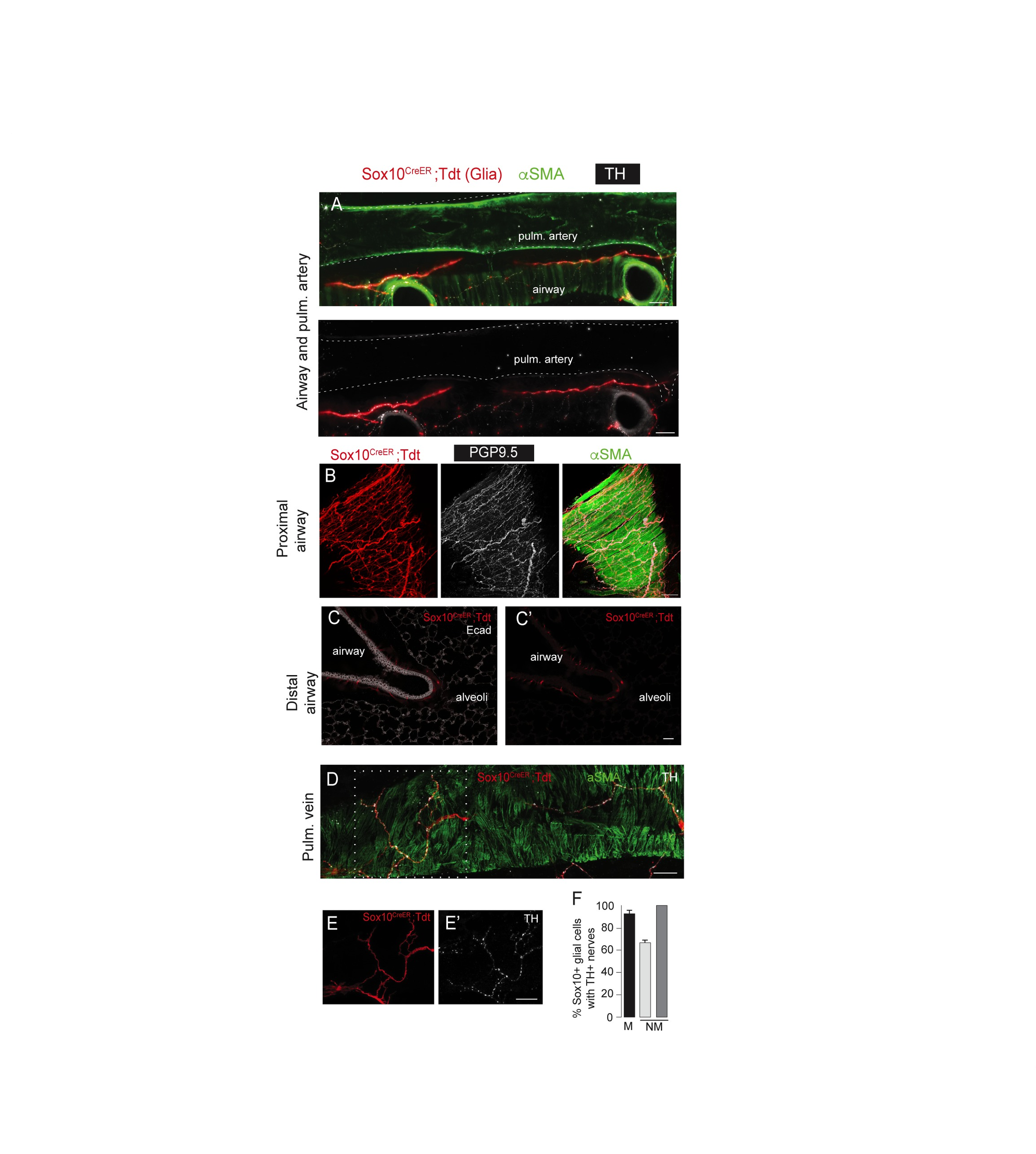

Supplement: S3 Fig — (A) Fluorescence microscope image of a proximal airway (computationally cleared, THUNDER Imager, Leica). Sox10-lineage-labeled glial cells (Sox10CreER > Tdt) form a mesh-like network along airways, but are absent from pulmonary arteries. Scale bar, 100 μm. αSMA, alpha smooth muscle actin. Pulmonary artery (pulm. artery) (B) Representative confocal image of a second airway showing NMG cells forming a networks along the airway surface closely associated with nerve fibers (protein gene produce 9.5/PGP9.5, white). Airway smooth muscle fibers (alpha smooth muscle actin/α-SMA, green). Scale bar, 100 μm (C) Representative sagittal section of distal airway surrounded by a network of Sox10-expressing glia (Sox10CreER>Tdt) and adjacent alveolar regions. Ecad, E-cadherin. Scale bar, 50 μm. (D) Confocal image of a representative section of pulmonary vein (pulm. vein) with Sox10CreER; Tdt labeled glial cells co-localizing with TH+ neuronal fibers. Alpha-smooth muscle actin, α−SMA. Bar, 50 μm. (E and E’) Individual channels highlighting boxed region in panel B showing Sox10CreER>Tdt lineage labeled NMG cells as individual channels shown for boxed region. TH, tyrosine hydroxylase. Bar, 50 μm. (F) Quantification of Sox10+ glial cells associated with TH+ nerves in myelinating (n = 247/267, 92.5% cells), non-myelinating (n = 1081/1623, 66.6%) airway glial cells vs. glial cells (n = 83/83, 100%) of pulmonary veins. Statistically significant differences were observed between airway MG vs. NMG cells (p < 0.0001) and between non-myelinating glial (NMG) cells of airway (A) vs. pulmonary vein (PV) (p < 0.0001). Two-proportion z-test. Error bars, 95% C.I. indicated. (TIF) [file pone.0310303.s003.tif]

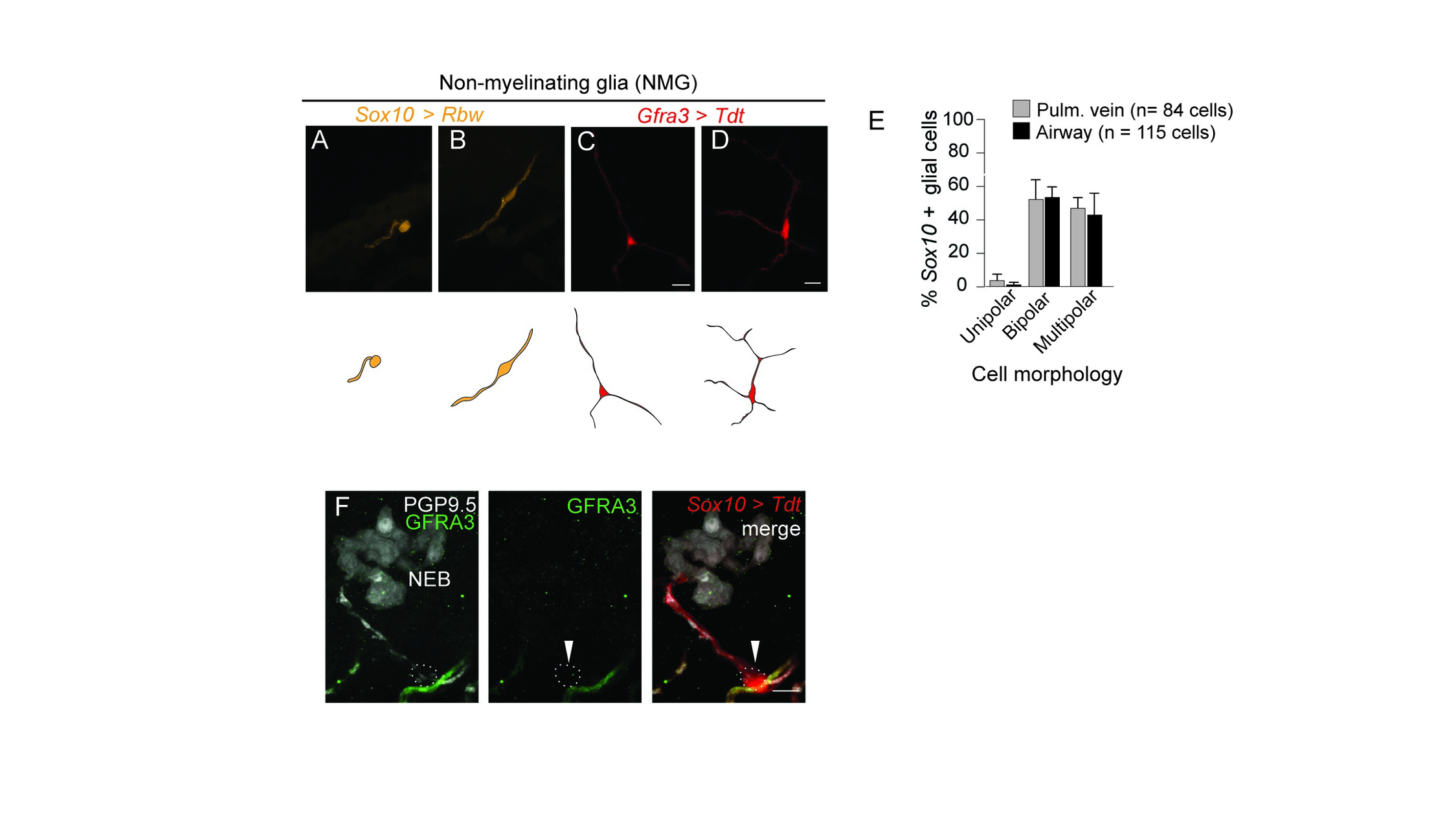

Supplement: S4 Fig — (A-B) Labeling of single non-myelinating glial cells along the airway using Sox10CreER mice in combination with the Rosa26LSL-Rbw multicolored reporter (Sox10CreER > Rbw). Two individual NMG cells along the airway representing (A) unipolar and (B) bipolar cells and their projections. Scale 10 μm. (C-D) Single multipolar glial cells labeled using Gfra3 CreER; Rosa26LSL-Tdt mice. Scale 10 μm. Sparse cell labeling was observed in glial cells with Gfra3CreER driving recombination or cells expressing the Rosa26LSL-Rbw reporter, which permitted visualization of single cell morphologies. Schematics showing fine extensions corresponding to each panel (below) (E) Quantification of Sox10 lineage+ glial cells by morphology of unipolar (n = 3/84 cells in pulmonary vein; 1/115 cells in airway), bipolar (45/84 cells in vein; 60/115 cells in airway), and multipolar (36/84 cells in vein; 54/115 cells in airway). Age of mice: PN 78 and 101 days, n = 2 mice. Grey bars, pulmonary vein (Pulm.vein); black bars, airway. Statistically significant differences were observed between unipolar vs bipolar, airway (p<0.0001), unipolar vs multipolar, airway (p<0.0001), unipolar vs bipolar, vein (p<0.0001), and unipolar vs multipolar, vein (p<0.0001). 2-proportion z test. Error bars, 95% C.I. (F) Basal TGNS cell in Sox10CreER > Tdt mice with TGNS soma (indicated by white arrowhead and outlined by white dotted lines) associated with a neuroepithelial body (NEB) and lacks GFRA3 expression (green) by immunohistochemistry. PGP9.5 (NEBs and neurons, white). Scale 10 μm. (TIF) [file pone.0310303.s004.tif]

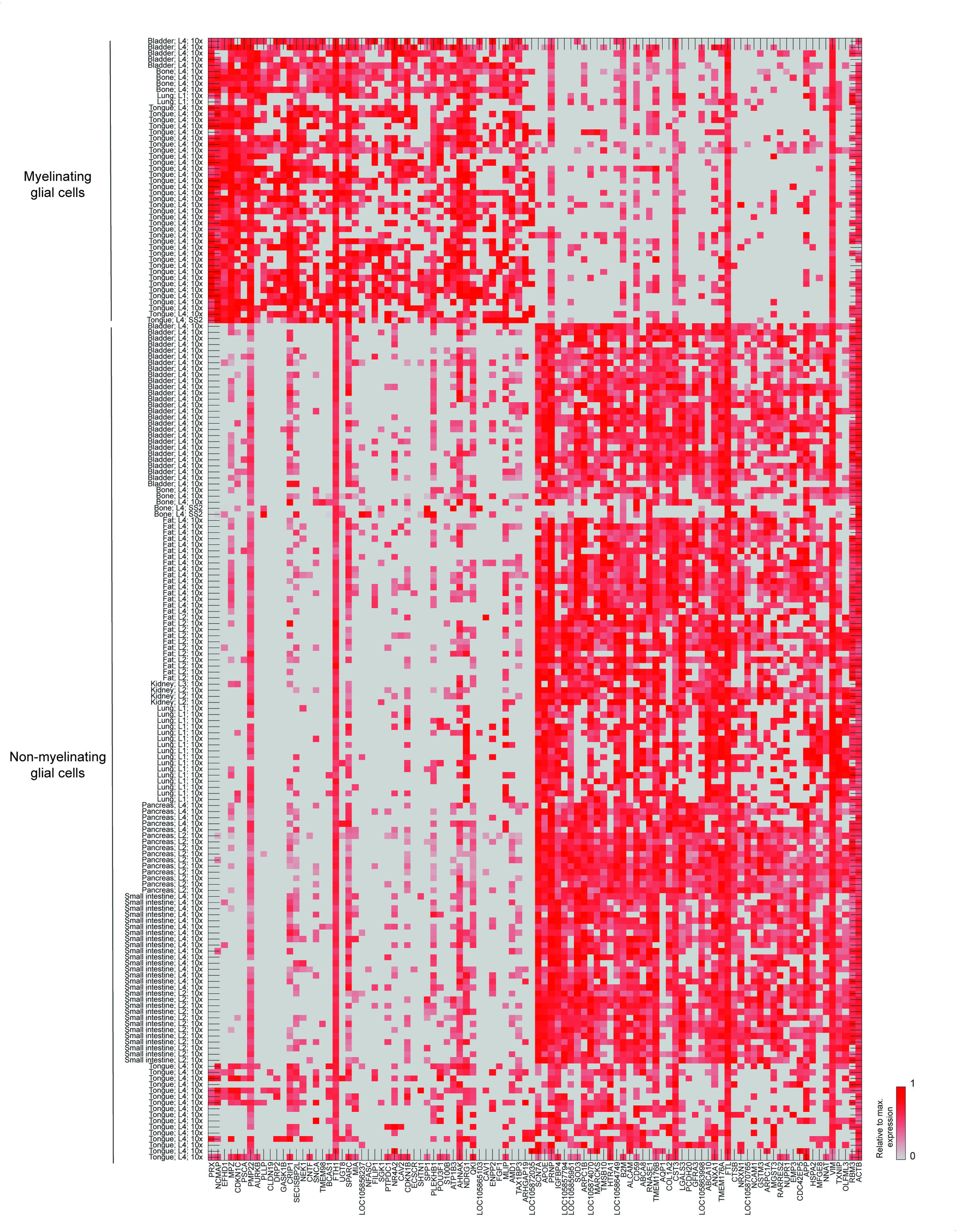

Supplement: S5 Fig — (A) Heatmap showing scRNA-seq expression levels of differentially expressed genes in the myelinating and non-myelinating glia identified in bladder, bone, lung, tongue, fat, kidney, pancreas, and small intestine from Tabula Microcebus Murinus [13]. The glial cells in Tabula Microcebus Murinus were annotated by expression of classic glial genes, recently reported genes in peripheral glia, and those we identified in our analysis of Tabula Muris Senis data. Relative expression levels are shown, normalized to the robust maximal (max.) (99th percentile) expression of the gene among all glia cell types. (TIF) [file pone.0310303.s005.tif]

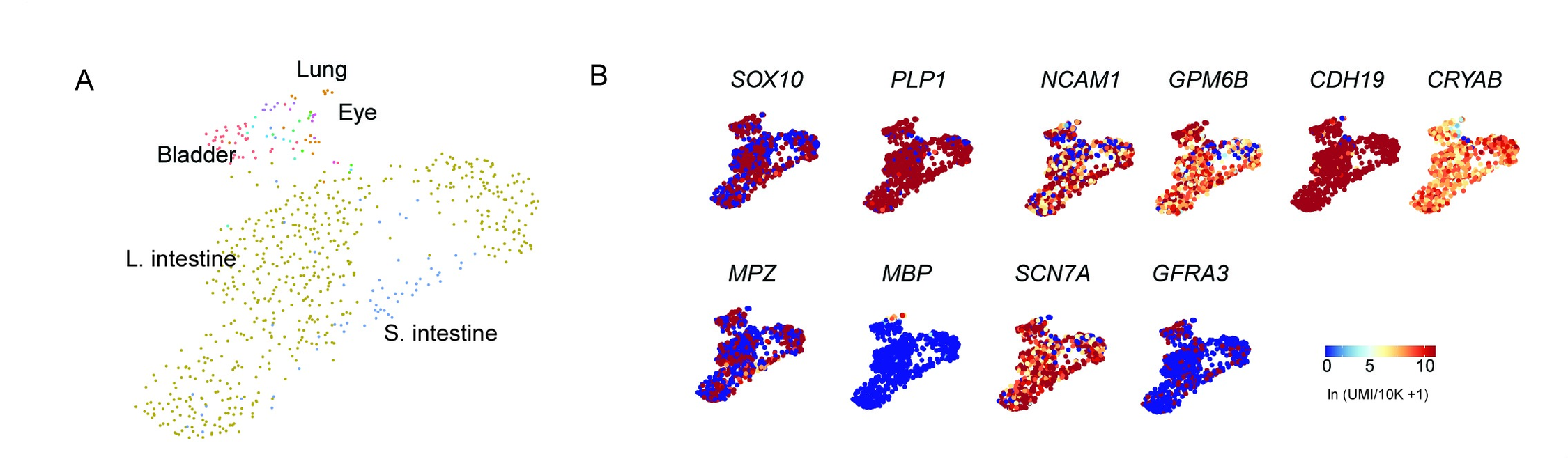

Supplement: S6 Fig — (A) UMAP plots representing glial cells isolated from each organ prior to filtering out the intestinal glial cells as previously described (also see methods) (B) Expression of pan-glial genes (SOX10, PLP1, NCAM1, GPM6B, CDH19, and CRYAB), and conserved expression of non-myelinating gene (SCN7A). (TIF) [file pone.0310303.s006.tif]

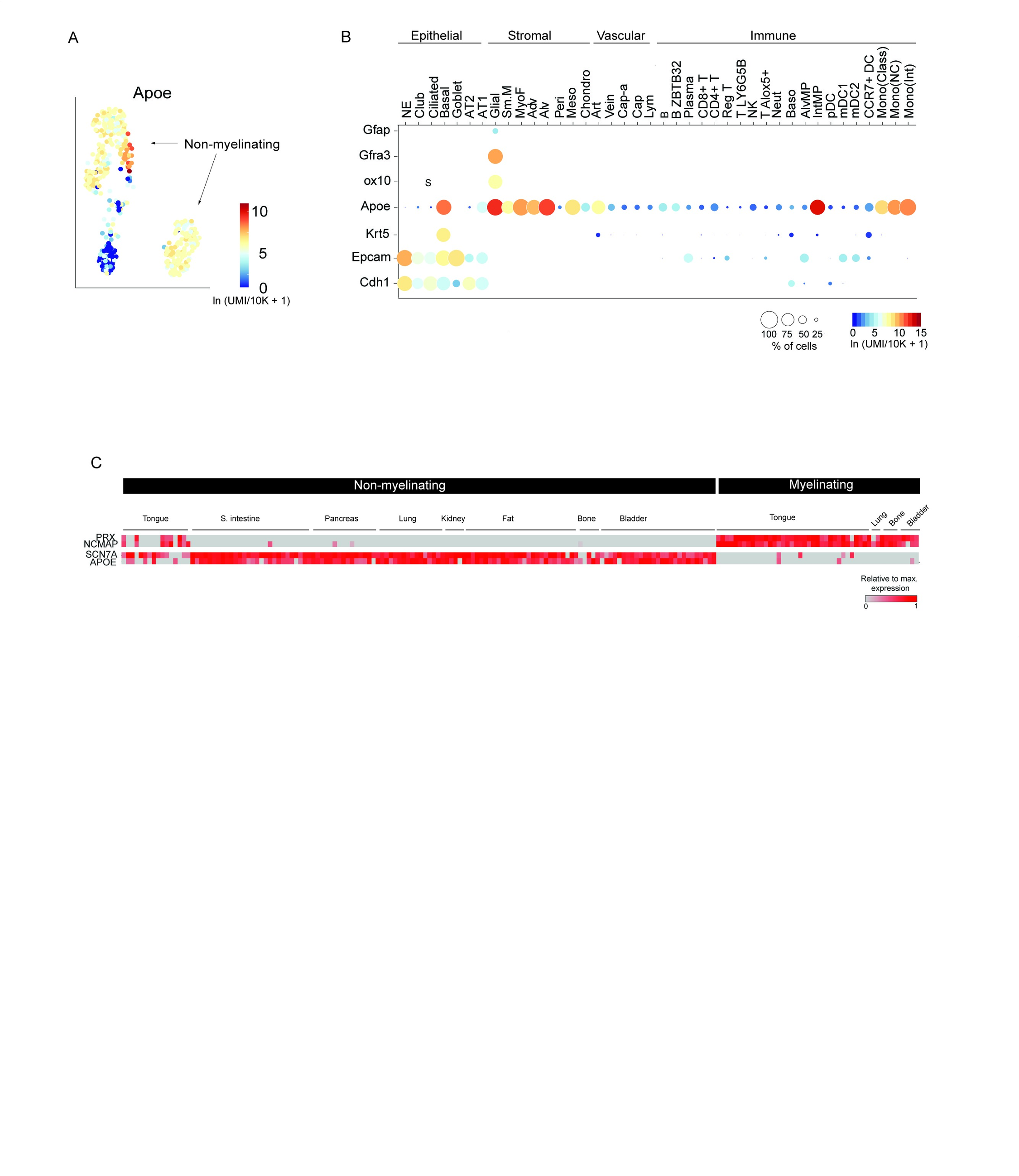

Supplement: S7 Fig — (A) UMAP representation of glial cells identified in mouse atlas with myelinating and non-myelinating glial cells from 7 different organs (Fig 3B and 3C). Scale. Values are log transformed unique molecular identifiers (UMI) per 10,000, ln (UMI/10K +1) (B) Dot plot showing expression of Apoe expression by scRNA-seq across all cells of the mouse lung cell atlas [15] across all major compartments (epithelial, stromal, vascular, and immune). Low level Apoe expression was observed in AT2 cells, and within the conducting airway epithelium, only basal cells expressed Apoe under basal conditions. Prominent expression within interstitial macrophages populations was observed. In experimental mouse models of allergic inflammation and ex vivo assays of human macrophages isolated from bronchoalveolar fluid, APOE secretion could be induced in a predominantly alveolar macrophage population [33]. Our current analysis reveals minimal expression in alveolar macrophages under basal conditions, suggesting an alternate molecularly defined population of macrophages that expresses APOE under basal conditions. Expression of glial markers (Gfap, Gfra3, Sox10). Expression levels of epithelial genes keratin 5/Krt5, EPCAM/Epcam and Cadherin-1/Cdh1 are shown. (C) Heatmap showing conserved expression of APOE in mouse lemur glial cells across the 8 organs represented (tongue; small intestine; S.intestine; pancreas; lung; kidney; fat; bone; and bladder). Relative expression levels are shown, normalized to the robust maximal (99th percentile) expression of the gene among all glia cell types. (TIF) [file pone.0310303.s007.tif]

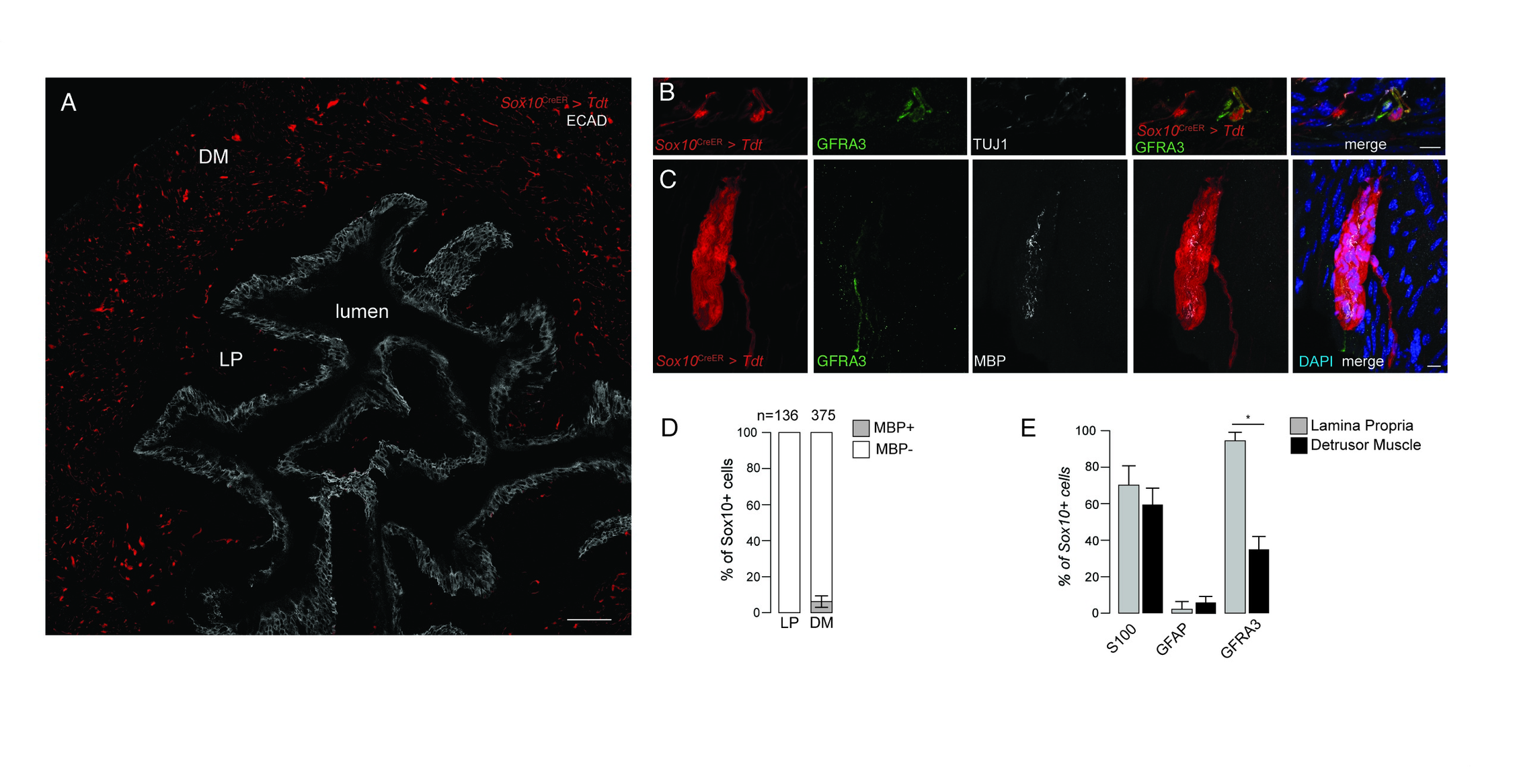

Supplement: S8 Fig — (A) Single plane cross-section through bladder showing prominent glial network in the outer detrusor muscle (DM) layer and a sparser network in the subepithelial lamina propria (LP) layer. E-cadherin (ECAD) expression is detected by fluorescence immunohistochemistry. Bar, 100 μm. (B) Close-up confocal single plane images of two non-myelinating Sox10 lineage positive (Sox10CreER > Tdt) glial cells in the detrusor muscle. Bar, 10 μm. (C) Close up images of myelinating glial cells running parallel to nerve fibers at the periphery of the bladder. Myelinated nerve fibers expressing myelin basic protein (MBP) indicated in adjacent panel. Bar, 10 μm. Note the absence of GFRA3 expression in the myelinating cells consistent with the scRNA-seq data. (D) Quantification of lineage-labeled (Sox10CreER > Tdt) glial cells co-expressing myelinating glial marker, myelin basic protein (MBP) in the detrusor muscle (DM) vs. the lamina propria (LP). MBP production by IHC: lamina propria (0/136, 0%); detrusor muscle (41/375, 11%). A statistically significant difference between the lamina propria and the detrusor muscle was observed (p<0.0001, 2 proportion z test). Error bars, 95% C.I. indicated. (E) Quantification of lineage-labeled (Sox10CreER > Tdt) glial cells co-expressing classic glial markers (S100B, GFAP) and GFRA3 in the detrusor muscle (DM) vs. the lamina propria (LP). S100 expression by immunohistochemistry (IHC): lamina propria (43/62, 69%); detrusor muscle (113/190, 59%). GFAP: lamina propria (1/46, 2%); detrusor muscle (13/210, 6%). GFRA3: lamina propria (78/83, 94%); detrusor muscle (69/186, 37%). A statistically significant difference between the lamina propria and the detrusor muscle was observed in GFRA3 (p<0.0001, 2 proportion z test). Error bars, 95% C.I. indicated. (TIF) [file pone.0310303.s008.tif]
