# Supplementary material for: Cellular and molecular characterization of peripheral glia in the lung and other organs
Source: PLoS One. 2024 Dec 2;19(12):e0310303. doi: 10.1371/journal.pone.0310303 (PMC11611111; doi:10.1371/journal.pone.0310303)
Supplement: S11 Table — (DOCX) [file pone.0310303.s019.docx]

**S11 Table. Summary of peripheral glial cell classes identified across organs and species**

|  | **Non-myelinating** | | | **Myelinating** | | | **TG** | | | **Terminal ^NS^** | | | **Satellite** | | |
| --- | --- | --- | --- | --- | --- | --- | --- | --- | --- | --- | --- | --- | --- | --- | --- |
| Lung | # | Morph. | Markers* | # | Morph. | Markers | # | Morph. | Markers | # | Morph. | Markers | # | Morph. | Markers |
| M | 11 | + (Fig2) | GFRA3 (Fig1)  *Gfra3*  *Scn7a*  *Gfra3* (Fig2) | 1 | + (Fig2) | *MBP** | 0 | - | - | 0 | + (Fig2) | - | 0 | + (Fig2) | - |
| L | 16 | - | *SCN7A*  *APOE*  *PRNP*  *IGFBP4*  *LGALS3*  *PCDH20*  ABCA10 (FigS6) | 2 | - | *NCAMP*  *MPZ*  *PLLP*  *PRX*  *EFHD1*  *CDKN1C*  *PSCA*  *AURKB*  (FigS6) | 0 | - | - | 0 | - | - | 0 | - | - |
| H | 2 | + (Fig2) | *GFRA3*  *SCN7A* (FigS7)  *NGFR*  *SCN7A*  *CDH2*  *L1CAM*  *NCAM1*  (FigS7) | 0 | - | *NAFSC*  *NCAMP*  *MBP*  *PRX5/3/23* | 0 | - | - | 0 | - | - | 0 | - | - |
| Bladder |  |  |  |  |  |  |  |  |  |  |  |  |  |  |  |
| M | 15 | + (Fig5) | *GFRA3**  *Scn7a*  *Gfra3*  *Ncam1*  *S100b*  (Fig2) | 0 | + (Fig5) | *MBP* | 0 | - | - | 0 | - | - | 0 | - | - |
| L | 27 | - | *SCN7A*  *APOE*  *PRNP*  *IGFBP4*  SOD3  ARPC1B  *LGALS3*  *PCDH20*  *ABCA10* (FigS6) | 5 | - | *NCAMP*  *MPZ*  *PLLP*  *PRX*  *EFHD1*  *CDKN1C*  *PSCA*  *AURKB* (FigS6) | 0 | - | - | 0 | - | - | 0 | - | - |
| H | 35 | - | *GFRA3* (FigS7) | 0 | - | *CLDN19*  *PRX*  *PLLP*  *BCAS1*  *MBP* (FigS7) | 0 | - | - | 0 | - | - | 0 | - | - |
| Limb m. |  |  |  |  |  |  |  |  |  |  |  |  |  |  |  |
| M | 87 | + | *Scn7a*  *Gfra3* (Fig3) | 34 | - | *Kcnj1*[1]  *Kir4*  *Cdh19*  *Fabp7*  *Ppara*  *Aldoc*  Mpz  *Prx*  *Pllp*  *Bcas1*  *Mbp*  (Fig2) | 137 | (+)[2] | *Bche*[2,3]  *Tinagl1*  *Gpc3*  *Cpm*  *Pla2g7*  *Kcnn4*  *Igfbp4*  *Sod3* (Fig2) | 0 | - | - | 0 | - | - |
| L | 0 | - | - | 0 | - | - | 0 | - | - | 0 | - | - | 0 | - | - |
| H | 0 | - | - | 0 | - | - | 0 | - | - | 0 | - | - | 0 | - | - |
| Heart |  |  |  |  |  |  |  |  |  |  |  |  |  |  |  |
| M | 70 | (+)[4] | S100  *Sox10+ lineage*  *Scn7a*  *Gfra3*  *Apoe* (Fig2) | 3 | (+) | *Mpz*  *Mbp*  *Pmp22*  *Prx*  *Pllp*  *Bcas1*  (Fig2) | 0 | - | *Tinagl1*  *Gpc3*  *Bche*  *Cpm*  *Pla2g7*  *Igfbp4*  *Sod3* (Fig2) | 0 | - | - | 0 | (+)[5] | S100[5]  B-FABP[5] (*Fabp*7) |
| L | 0 | - |  | 0 | - | - | 0 | - | - | 0 | - | - | 0 | - | - |
| H | 0 | - | - | 0 | - | - | 0 | - | - | 0 | - | - | 0 | - | - |
| Trachea |  |  |  |  |  |  |  |  |  |  |  |  |  |  |  |
| M | 31 | - | *Scn7a*  *Igfbp7*  *Gfra3*  *Apoe* (Fig2) | 10 | - | *Cldn19*  *Prx*  *Pllp*  *Mbp*  *Mpz* | 3 | - | - | 0 | - | - | 0 | - | - |
| L | 0 | - | - | 0 | - | - | 0 | - | - | 0 | - | - | 0 | - | - |
| H | 0 | - | - | 0 | - | - | 0 | - | - | 0 | - | - | 0 | - | - |
| Fat |  |  |  |  |  |  |  |  |  |  |  |  |  |  |  |
| M | 10 | - | *P75NTR*[6]  *Scn7a*  *Gfra3* (Fig2) | 12 | (-)^44^ | Plp1[7]  MBP[6]  MPZ[6]  *Pmp22*  *Cldn19*  *Prx*  *Pllp*  *Bcas1*  *Mbp*  *Mpz* | 3 | - | *Gpc3*  *Bche*  *Cpm*  *Pla2g7*  (Fig2) | 0 | - | - | 0 | - | - |
| L | 27 | - | *SCN7A*  *APOE*  *PRNP*  *IGFBP4*  *SOD3*  *ARPC1B*  *LGALS3*  *PCDH20*  *ABCA10*  *GFRA3* (FigS3) | 0 | - | *NCAMP*  *MPZ*  *PLLP*  *PRX*  *EFHD1*  *CDKN1C*  *PSCA*  *AURKB* (FigS3) | 0 | - | - | 0 | - | - | 0 | - | - |
| H | 0 | (+)^46^ | S100[8] | 0 | - | *-* | 0 | (+)^46^ | S100^46^ | 0 | - | - | 0 | - | - |
| Kidney |  |  |  |  |  |  |  |  |  |  |  |  |  |  |  |
| M | 8 | - | S100[9]  *Scn7a*  *Gfra3*  *Apoe* (Fig2) | 0 | - | - | 0 | - | - | 0 | - | - | 0 | - | - |
| L | 4 | - | - | 0 | - | - | 0 | - | - | 0 | - | - | 0 | - | - |
| H | 0 | - | - | 0 | - | - | 0 | - | - | 0 | - | - | 0 | - | - |
| Pancreas |  |  |  |  |  |  |  |  |  |  |  |  |  |  |  |
| M | 0 | (+)[10] | GFAP[10]  S100B11/15/23 5:31:00 PM | 0 | - | MPZ^49^ | 0 | - | - | 0 | - | - | 0 | - | - |
| L | 15 | - | *SCN7A*  *APOE*  *PRNP*  *IGFBP4*  *SOD3*  *ARPC1B*  *LGALS3*  *PCDH20*  *ABCA10*  *GFRA3*  (FigS3) | 0 | - | - | 0 | - | - | 0 | - | - | 0 | - | - |
| H | 4 | - | GFAP  *APOE* (FigS7) | 0 | - | - | 0 | - | - | 0 | - | - | 0 | - | - |
| Sm. int. | # | Morph. | Markers | # | Morph. | markers | # | Morph. | markers | # | Morph. | markers | # | Morph. | markers |
| M | 0 | (+)[11] | Dhh-lineage and Sox10[11] | 0 | - | - | 0 | - | - | 0 | - | - | 0 | - | - |
| L | 28 | - | *SCN7A*  *APOE*  *PRNP*  *IGFBP4*  SOD3  ARPC1B  *LGALS3*  *PCDH20*  *ABCA10* | 0 | - | - | 0 | - | - | 0 | - | - | 0 | - | - |
| H | 1 | - | - | 0 | - | - | 0 | - | - | 0 | - | - | 0 | - | - |
| Bone |  |  |  | # |  |  |  |  |  |  |  |  |  |  |  |
| M | 0 | - | - | 0 | - | - | 0 | - | - | 0 | - | - | 0 | - | - |
| L | 5 | - | *SCN7A* (FigS3)  *APOE* (FigS3)  *PRNP* (FigS3) | 4 | - | - | 0 | - | - | 0 | - | - | 0 | - | - |
| H | 0 | - | - | 0 | - | - | 0 | - | - | 0 | - | - | 0 | - | - |
| Eye |  |  |  |  |  |  |  |  |  |  |  |  |  |  |  |
| M | 0 | - | - | 0 | - | - | 0 | - | - | 0 | - | - | 0 | - | - |
| L | 1 | - | - | 0 | - | - | 0 | - | - | 0 | - | - | 0 | - | - |
| H | 14 | - | *APOE*  *GFRA3*  *IGFBP7* (FigS7) | 0 | - | *CLDN19*  *PRX*  *PLLP*  *BCAS1*  *MBP* (FigS7) | 0 | - | - | 0 | - | - | 0 | - | - |
| Thymus |  |  |  |  |  |  |  |  |  |  |  |  |  |  |  |
| M | 0 | + | GFAP[12] | 0 | - | - | 0 | - | - | 0 | - | - | 0 | - | - |
| L | 0 | - | - | 0 | - | - | 0 | - | - | 0 | - | - | 0 | - | - |
| H | 7 | - | *GFRA3* (FigS7) | 0 | - | *CLDN19*  *PRX*  *PLLP*  *BCAS1*  *MBP* (FigS7) | 0 | - | - | 0 | - | - | 0 | - | - |
| Tongue |  |  |  |  |  |  |  |  |  |  |  |  |  |  |  |
| M | 0 | - | - | 0 | - | - | 0 | - | - | 0 | - | - | 0 | - | - |
| L | 28 | - | *SCN7A*  *APOE*  *IGFBP4*  *LGALS3* (FigS3) | 36 | - | - | 0 | - | - | 0 | - | - | 0 | - | - |
| H | 0 | - | - | 0 | - | - | 0 | - | - | 0 | - | - | 0 | - | - |
| Prostate |  |  |  |  |  |  |  |  |  |  |  |  |  |  |  |
| M | 0 | + | - | 0 | - | - | 0 | - | - | 0 | - | - | 0 | - | - |
| L | 0 | - | - | 0 | - | - | 0 | - | - | 0 | - | - | 0 | - | - |
| H | 3 | - | S100[13]  *GFRA3* (FigS7) | 0 | - | - | 0 | - | - | 0 | - | - | 0 | - | - |
| S.gland |  |  |  |  |  |  |  |  |  |  |  |  |  |  |  |
| M | 0 | - | - | 0 | - | - | 0 | - | - | 0 | - | - | 0 | - | - |
| L | 0 | - | - | 0 | - | - | 0 | - | - | 0 | - | - | 0 | - | - |
| H | 2 | - | - | 0 | - | - | 0 | - | - | 0 | - | - | 0 | - | - |

M, mouse; L, lemur; H, human. #, number of glial cells identified by scRNA-seq, NMJ, neuromuscular junction; NS, neurosensory;

Sm.int. (small intestine); S.gland (salivary gland)

*Genes are italicized; in cases where expression confirmed by immunohistochemistry, the protein name is listed (non-italicized).

Supporting evidence either from a figure in current article or from prior reports with reference cited.

Note: *S100b*-expressing Schwann cells reported in rat salivary glands^53^

Note: Genes for lemur glial subtypes were selected from S6 Fig and included if they were expressed in > 75% of the target subtype

and < 25% of non-glial cells

Pan-glial genes identified in *Tabula Muris Senis*: *Sox10, Plp1, Gpm6b, Kcna1, Cryab, Prnp*

Pan-glial genes in *Tabula Microcebus*: *SOX10*, *PLP1, GPM6B, CRYAB*

Pan-glial genes in *Tabula Sapiens*: *SOX10*, *PLP1, GPM6B, CDH19, CRYAB*

**References (Supplemental table)**

1. Proietti D, Giordani L, De Bardi M, D’Ercole C, Lozanoska-Ochser B, Amadio S, et al. Activation of skeletal muscle–resident glial cells upon nerve injury. JCI Insight. 2021 Apr 8;6(7):e143469.

2. Castro R, Taetzsch T, Vaughan SK, Godbe K, Chappell J, Settlage RE, et al. Specific labeling of synaptic schwann cells reveals unique cellular and molecular features. Stevens B, Westbrook GL, Ko CP, editors. eLife. 2020 Jun 25;9:e56935.

3. Ko CP, Robitaille R. Perisynaptic Schwann Cells at the Neuromuscular Synapse: Adaptable, Multitasking Glial Cells. Cold Spring Harb Perspect Biol. 2015 Oct;7(10):a020503.

4. Hortells L, Meyer EC, Thomas ZM, Yutzey KE. Periostin-expressing Schwann cells and endoneurial cardiac fibroblasts contribute to sympathetic nerve fasciculation after birth. Journal of Molecular and Cellular Cardiology. 2021 May;154:124–36.

5. Fregoso SP, Hoover DB. Development of cardiac parasympathetic neurons, glial cells, and regional cholinergic innervation of the mouse heart. Neuroscience. 2012 Sep 27;221(C):28–36.

6. Willows JW, Gunsch G, Paradie E, Blaszkiewicz M, Tonniges JR, Pino MF, et al. Schwann cells contribute to demyelinating diabetic neuropathy and nerve terminal structures in white adipose tissue. iScience. 2023 Mar;26(3):106189.

7. Stavely R, Hotta R, Picard N, Rahman AA, Pan W, Bhave S, et al. Schwann cells in the subcutaneous adipose tissue have neurogenic potential and can be used for regenerative therapies. Sci Transl Med. 2022 May 25;14(646):eabl8753.

8. Fede C, Petrelli L, Pirri C, Neuhuber W, Tiengo C, Biz C, et al. Innervation of human superficial fascia. Front Neuroanat. 2022 Aug 29;16:981426.

9. Darlot F, Artuso A, Lautredou-Audouy N, Casellas D. Topology of Schwann cells and sympathetic innervation along preglomerular vessels: a confocal microscopic study in protein S100B/EGFP transgenic mice. American Journal of Physiology-Renal Physiology. 2008 Oct;295(4):F1142–8.

10.Sunami E, Kanazawa H, Hashizume H, Takeda M, Katsuyoshi H, Ushiki T. Morphological characteristics of Schwann cells in the islets of langerhans of murine pancreas. Arch Histol Cytol. 2001;64(2):191–201.

11.Uesaka T, Okamoto M, Nagashimada M, Tsuda Y, Kihara M, Kiyonari H, et al. Enhanced enteric neurogenesis by Schwann cell precursors in mouse models of Hirschsprung disease. Glia. 2021 Nov;69(11):2575–90.

12.Hu D, Nicholls PK, Yin C, Kelman K, Yuan Q, Greene WK, et al. Immunofluorescent Localization of Non-myelinating Schwann Cells and Their Interactions With Immune Cells in Mouse Thymus. J Histochem Cytochem. 2018 Nov;66(11):775–85.

13.Shapiro E, Seller J, Lepor H, Kalousek K, Hutchins GM, Perlman EJ, et al. Altered smooth muscle development and innervation in the lower genitourinary and gastrointestinal tract of the male human fetus with myelomeningocele. The Journal of Urology. 1998;1047–63.
