# Supplementary material for: Cellular and molecular characterization of peripheral glia in the lung and other organs
Source: PLoS One. 2024 Dec 2;19(12):e0310303. doi: 10.1371/journal.pone.0310303 (PMC11611111; doi:10.1371/journal.pone.0310303)
Supplement: Attachment — Submitted filename: Response to Reviewers.pdf [file pone.0310303.s022.pdf]

August 19, 2024

Dear Dr. Tanigaki,
Thank you for handling our manuscript and for the constructive comments and suggestions. Please see our revisions and responses (bold type) to each point below.

Response to Reviewer comments and questions

Reviewer #1

(1) In Figure 5C Tabula Microcebus data (mouse lemur), pan-glial markers seem to be weak, compared with the mouse and human data (the same heatmaps in Figure 2I and Figure 5H. Could the authors briefly explain the reasons?

Thank you for noting this. In the original submission Fig 5C, the plotted data values were scaled after log normalization and this has been corrected. In this version, the more highly-expressed pan-glial markers (*PLP1*, *GPM6B*, *PRNP*, and *CRYAB*) are broadly expressed across the glial population. The proper scale values for the corresponding color bar are shown. The overall differences in expression level may reflect either technical variations or underlying biological differences. While we cannot be certain if the lower expression level is primarily technical, we think that differences in sample preparation or single cell RNA sequencing (scRNA-seq) methodology could explain the lower total number of reads/cell in the Tabula Microcebus Murinus (lemur) glial cell data, thus potentially increasing the gene 'drop-out' rate in the scRNA-seq data.

To clarify this point, we have added additional analysis in the corresponding figure legend for Fig. 5 (pg. 17-18) as follows:

"The gene expression level for pan-glial markers is lower in the Tabula Microcebus data compared to Tabula Muris Senis and Tabula Sapiens data sets. These differences may reflect technical variations in sample preparation for scRNA-seq. Lower # read counts/glial cell in the lemur data (median: 2112; 10th – 90th percentile range: 1097-5430 reads/cell) could result in higher gene "drop out" rate compared to mouse (median: 3715 reads/cell; 10th – 90th percentile range: 2691-229491 reads/cell) and human data (median: 4155 reads/cell; 10th – 90th percentile range: 2844-18263 reads/cell). The number of read counts per glial cell are listed in S2 and S5 Tables for mouse and human, respectively. Lemur read counts for each glial cell included in S6 Table are previously reported (Tabula Microcebus Consortium, 2021.
bioRxiv 2021.12.12.469460; doi: https://doi.org/10.1101/2021.12.12.469460.

(2) In Figure 1E: please label all cell types in the violin plots. It is up to the authors to see if it is also useful to include marker genes for other cell types.

We have edited and included Fig. 1D and E to annotate all the other cell types and included violin plots showing representative marker gene expression for each cell type. We previously described the isolation scheme, filtering and processing of scRNA-seq data for all the clusters and emphasized neuroendocrine cell analysis (Kuo et al, eLife 2022). Here, we analyzed the glial cell cluster.

(3) In Figure 2/5/6 and many supp figures: please clarify gene expression scale. "ln(UMI/K+1)" shows up in many main figures, "ln(UMI/10K+1)" shows up in many supplementary figures, and "ln(CP10K+1)" shows up sometimes. According to the text description, "ln(UMI/K+1)" may not be correct. The other two should mean the same thing, and either one is fine.

Thank you for identifying this issue. Yes, we intended for these to designate the same expression scale and have resolved the inconsistencies and changed all the appropriate legends to "ln (UMI/10K + 1)".

(4) Some supplementary tables are off — they need to be correctly ordered and referenced. Below are a few (maybe incomplete) instances:

-In Methods “p-values from the rank-sum test were then Bonferroni adjusted and genes with adjusted p-value < 0.05 were shown in Table X”
-“Next, we applied Portal [29] to integrate the data and embed the data to a species-integrated UMAP (Table S7)”, which should be table S6?
“Genes with a Bonferroni corrected p-value smaller than 0.05 and a 3-fold increased expression were selected and listed in Table S6.”, which does not contain DEGs information.
-In the Supplementary table list “S2 Table. Differentially expressed glial genes in mouse peripheral glial (Tabula Muris Senis)”, which should be Table S3?

Thank you identifying these mislabeled supplemental tables and we apologize for any confusion. We have corrected the table titles and referencing within the text for each of these. We also edited the remaining tables to confirm accurate citation. Note: we adjusted the order of S8 and S9 Tables to match the new order of feature plots presented in Fig. 6. We made these adjustments to highlight the differential gene expression analysis performed for the three molecular groups of glial cells as suggested by reviewer #2.

Reviewer #2
(1) Figure 1: It would be great if the authors could provide a more comprehensive confocal image showing labeling of Ascl1;Rosazsgreen in the lung, perhaps a low-magnification image.

Thank you for this suggestion. We added S1 Fig (panels B - C) to show both a low-magnification image of a similar lineage-labeled lung sample from an *Ascl1 > Zsgreen1* mouse lung and a detailed description of the figure on p. 32. Glial cell progenitors that transiently express the transcription factor, *Ascl1*, during development are indicated (green arrowheads). Because they are sparsely distributed, we show a representative cryosection through an entire lobe of mouse lung to demonstrate both glial and neuroendocrine progenitors are labeled. Please note that neuroendocrine cells are epithelial and can be distinguished by co-expression of E-cadherin (as we previously showed in Kuo et al (2022 eLife).

(2) Figures 1D-E: Please also show the expression patterns of canonical markers for other cell types in the feature plots or violin plots.

We have included violin plots (Fig. 1, panel G) showing expression of classic markers that were differentially expressed in each of the other 9 cell types. Our single cell sorting/gating method and subsequent scRNA-seq processing for this data set was previously described in our study focusing on pulmonary neuroendocrine cells (Kuo et al, 2022 eLife). Fig. 1, panel F now shows each cluster colored to represent the corresponding cell type represented by the same shading in the violin plots in panel F.

(3) Figure 2A: This figure is not cited in the text, and it is unclear why the authors present the expression pattern of Plp1. Is it a pan-glial marker? It would make more sense to show the expression patterns of pan-glial markers in the atlas they analyzed in the main figure.

Thank you for these additional comments and suggestions. We have edited the text to clarify and added another classic pan-glial gene, *Sox10*, in Figure 2A to show it is also expressed at high levels within the glial cell-enriched cluster we selected for subsequent analysis. Since *Plp1* is one of the classic markers used to identify glial cells, we also presented its expression pattern to demonstrate our approach to identifying a cluster of 4300 cells within the whole organism scRNA-seq atlas enriched for glial cells.

From this cluster, we identified a final set of 435 peripheral glial cells. Our strategy to identify these is related to the next point and described more below. Please also note that some cells also expressed *Sox10* in the mammary gland (cluster #42), but those cells did not express the other glial markers and were thus not included in our peripheral glia dataset.

(4) Figure S2A-D: These figures are not cited. In Figure S2, please add a UMAP plot with the corresponding cluster numbers labeled. Also, the text states that 435 glial cells were found, composing 0.12% of the atlas. Why does it show 4300 cells, 1.2%, in Figure S2B?

Thank you for these suggestions and clarification questions. We have included additional text on pgs. 6-7 (and in methods section on pg. 28) to clarify the cell numbers and percentages by explaining our strategy for filtering the 4300 cells within the glial cell cluster (cluster 32, Fig. S2A) more clearly in the main text. We added a UMAP plot with the corresponding cluster numbers from the Tabula Muris Senis atlas (Fig. S2B) and replaced the prior UMAP figure showing only cluster 32 shaded.

We added the following explanation to the methods section (pg. 28):
"Of the 4300 cells in cluster #32 (which represents 1.2% of the total # cells in the entire atlas comprised of 356,213 cells), 3176 cells were from the central nervous system. After filtering these cells, which left us with 1,124 cells, we screened each of the remaining cells for expression of expanded gene panels of classic and newly identified glial cell genes to confirm glial cell identity. In addition, we searched for gene expression of other cell types that could be contaminants (stromal, melanocyte, and epithelial cells) and filtered out those cells (Fig. S2E-F). This left 435 cells of the total 355,213 (0.12%).

We also included the citations to Fig. S2A-D on pg. 6 of the manuscript.

(5) Differential Expression (DE) Analysis: It would be interesting to perform DE analysis among the three glial types and investigate their molecular signatures.

We agree this is important and performed this analysis initially using the Tabula Muris Senis data (Figs. 2, S2). We have also edited the corresponding Table S3 to more clearly present the top differentially expressed genes representing the three glial types identified by scRNA-seq analysis (myelinating, non-myelinating and terminal). In our cross-species analysis of peripheral glia, we integrated the scRNA-seq data from mouse, lemur, and human, to generate a dataset with 695 peripheral glial cells. We edited Fig. 6 and the supplemental tables (Tables S7-10) corresponding to the differential gene expression analysis using the integrated glia to show the three molecular types. The top genes are listed and the dataset with curated metadata made available.

(6) Gfra3 Expression: It is interesting to note that Gfra3 is selectively expressed in NMG. How about other growth factor receptors?
We agree this is an intriguing finding and have examined the remaining family members related to the glial cell derived neurotrophic factor (GDNF), receptor alpha 3 (Gfra3). The GDNF family ligands and receptors are important for neural development we plan to build on in subsequent studies. Here, we present additional feature plots showing expression of other known receptors in this family that transduce neurotrophic signals in peripheral glia from our integrated data set of peripheral glia across species. The other receptors in addition to GFRA3 are *RET* (receptor tyrosine kinase), *GFRA1*, *GFRA2*, *GFRA3*. The molecular clusters correspond to those in Fig. 6 and are shown here (panel A) and receptor expression is shown in panel B. Interestingly, *GFRA1* is the only GDNF receptor alpha gene expressed in terminal glial cells and it is not as broadly expressed in other *NMG*. *GFRA2* is also expressed in a subset of non-myelinating glia but not in terminal glia.

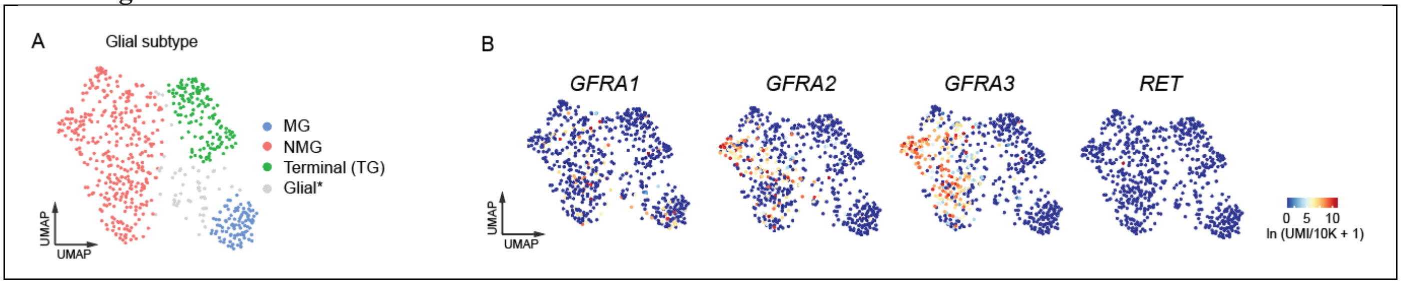

(7) Figure 6: Again, it would be interesting to see the molecular signatures of these three glial cell types.

Thank you for this feedback. We agree and have edited Fig. 6 to highlight some key molecular distinctions across the three glial cell types. We also included an additional category to identify expression of differentially expressed genes in the broader class of non-myelinating glia (including terminal cells) and highlighted the genes that were expressed in both of these classes but not myelinating glial cells (Fig. 6G). The full set of differentially expressed genes is reported for each of the three classes in Tables S7-S9 and the full set of genes distinguishing non-myelinating (including terminal glial) cells in Table S10.

(8) There are some grammar issues and typos. For instance, "we used the expression profiles from both the Tabula Muris Senis and Ascl1-enriched lung scRNA-seqdatasets described above to predicted that" -> "to predict that "Individual non-myelinating airway and vascular glial cells were often had multipolar projections compared to myelinating glia cells" -> "glial cells often had"

Thank you for identifying these typos. We have corrected them.
